# A virulence-associated small RNA MTS1338 activates an ABC transporter CydC for rifampicin efflux in *Mycobacterium tuberculosis*

**DOI:** 10.3389/fmicb.2024.1469280

**Published:** 2024-09-19

**Authors:** Saumya Singh, Tanmay Dutta

**Affiliations:** RNA Biology Laboratory, Department of Chemistry, Indian Institute of Technology Delhi, New Delhi, India

**Keywords:** small RNAs, gene regulation, antimicrobial resistance, efflux protein, drug efflux

## Abstract

The efficacy of the tuberculosis treatment is restricted by innate drug resistance of *Mycobacterial tuberculosis* and its ability to acquire resistance to all anti-tuberculosis drugs in clinical use. A profound understanding of bacterial ploys that decrease the effectiveness of drugs would identify new mechanisms for drug resistance, which would subsequently lead to the development of more potent TB therapies. In the current study, we identified a virulence-associated small RNA (sRNA) MTS1338-driven drug efflux mechanism in *M. tuberculosis*. The treatment of a frontline antitubercular drug rifampicin upregulated MTS1338 by >4-fold. Higher intrabacterial abundance of MTS1338 increased the growth rate of cells in rifampicin-treated conditions. This fact was attributed by the upregulation of an efflux protein CydC by MTS1338. Gel-shift assay identified a stable interaction of MTS1338 with the coding region of *cydC* mRNA thereby potentially stabilizing it at the posttranscriptional level. The drug efflux measurement assays revealed that cells with higher MTS1338 abundance accumulate less drug in the cells. This study identified a new regulatory mechanism of drug efflux controlled by an infection-induced sRNA in *M. tuberculosis*.

## 1 Introduction

Persistence of *Mycobacterial tuberculosis*, the aetiological agent of tuberculosis (TB), in the hostile environment of host macrophages relies on fast and precise reprogramming of transcriptional profile in response to external stimuli (Miotto et al., 2022; Arnvig and Young, 2012; Dutta and Srivastava, 2018; Taneja and Dutta, 2019). An array of complex regulatory networks controlled by transcriptional modulators, two-component systems, sigma factors, riboswitches, and small RNAs (sRNAs) fine-tune the mycobacterial gene expression for adaptation to external challenges in real-time (Miotto et al., 2022; Taneja and Dutta, 2019; Smith et al., 2013; Petchiappan and Chatterji, 2017), which render them notoriously difficult to eliminate from infected patients. Consequently, prolonged treatment with frequent administration of multiple antibiotics is required to treat TB (Ginsberg and Spigelman, 2007; Dhar and McKinney, 2010; van den Boogaard et al., 2009). *M. tuberculosis* exploits a plethora of intrinsic mechanisms to combat anti-TB drug therapy (Remm et al., 2022). Its complex hydrophobic cell envelope is impermeable to many drugs and is considered as one of the key features of defense against drug influx (Sarathy et al., 2012). *M. tuberculosis* possesses additional machinery for drug tolerance, which includes multiple efflux pumps that deport antimycobacterial compounds out of the cells, mycobacterial enzymes capable of chemical modification of antibiotics, and alterations in gene expression to adapt to antibiotic-exposed conditions (Briffotaux et al., 2019; Nasiri et al., 2017). Antibiotics also lead to the development of acquired *M. tuberculosis* resistance against it through target mutations (Takiff and Feo, 2015), which is associated with the reduced fitness of the resistant mutants (Andersson and Hughes, 2010). Because of the above-mentioned capabilities of *M. tuberculosis*, illegitimate drug usage by patients frequently causes a high degree of treatment failure leading to disease relapse and emergent drug resistance (Dhar and McKinney, 2010; van den Boogaard et al., 2009) triggering onward transmission and amplification of drug-resistant strain (Barilar and Battaglia, 2024).

Traditional antimycobacterial compounds interfere with the biosynthetic processes, e.g., biogenesis of cell wall, transcription, and protein synthesis, which are necessitated for cell growth (Dhar and McKinney, 2010). A few of these antibiotics like isoniazid, ethambutol, etc. for inhibition of cell wall formation, rifampicin for interference with transcription, and streptomycin for the inhibition of protein synthesis of *M. tuberculosis* are regarded as the frontline anti-TB drugs (Briffotaux et al., 2019). *M. tuberculosis* cells although highly susceptible to death in the presence of these antibiotics *in vitro*, are less affected by the same antimycobacterial agents while grown *in vivo* in mammalian hosts (Dhar and McKinney, 2010; Young et al., 2008; McKinney, 2000). Therefore, chronic TB is more difficult to control through a single antibiotic therapy and calls for second-line regimens. Patient nonadherence to such an extended therapeutical procedure, insufficient health infrastructure, and lack of antibiotics help promote the development of resistant strains of exceedingly slow-growing *M. tuberculosis* (Briffotaux et al., 2019). Hence, multidrug-resistant TB (MDR-TB) and extremely drug-resistant TB (XDR-TB) strains have evolved (Almeida Da Silva and Palomino, 2011; Udwadia et al., 2012) which accentuates the requirement for new strategies to develop novel anti-TB drugs. For that reason, it is essential to comprehensively understand the regulatory networks promoting resistance in *M. tuberculosis* against antibiotics.

Although sRNAs have been identified to be a ubiquitous class of regulators contributing to antibacterial resistance in pathogenic bacteria (Mediati et al., 2021), sRNA-mediated regulation of antibiotic resistance in *M. tuberculosis* has not been investigated yet. Only a handful of sRNAs among a few dozen discovered in *M. tuberculosis* until now have been identified with associated functions given the difficulty of understanding sRNA function *in vivo* in *M. tuberculosis* (Dutta and Srivastava, 2018; Arnvig et al., 2011; Singh et al., 2021). MTS1338 (or ncRv11733) is one among those sRNAs, which exists only in the pathogenic mycobacterial genome (Dutta and Srivastava, 2018; Arnvig et al., 2011; Moores et al., 2017), and is highly accumulated during *M. tuberculosis* infection (Arnvig et al., 2011). Consequently, MTS1338 is highly upregulated in response to stresses associated with infection (Singh et al., 2021; Salina et al., 2019) and configures a stress-resistance signature in *M. tuberculosis* (Martini et al., 2023). Substantial alteration of the transcriptomic profile of *M. tuberculosis* overexpressing MTS1338 suggests its potential role in the global gene regulatory network, which promotes the survival of *M. tuberculosis* in host macrophages through an adaptive mechanism (Martini et al., 2023). Participation of MTS1338 in the regulation of numerous physiological processes firmly indicated that MTS1338 might play an important role in antimycobacterial resistance.

In the current study, we identified that MTS1338 is highly induced while the cells are exposed to antibiotic like rifampicin. Cells with higher intracellular MTS1338 accumulation were ascertained to grow at a higher rate than the cells with basal MTS1338 level. Investigations on how MTS1338 in its higher abundance promotes *M. tuberculosis* growth affirmed that MTS1338 increases the abundance of the mRNA of an efflux protein CydC by binding to its coding region potentially protecting it at the posttranscriptional stage from cellular endoribonucleases. Inhibition of efflux proteins of the cells undergone a prior treatment with rifampicin resulted in a better mycobacterial growth rate for the cells overexpressing MTS1338. A higher abundance of intracellular MTS1338 resulted in a lower accumulation of antibiotics in the cell suggesting a potential role of this sRNA in dealing with mycobacterial adaptation to antibiotics.

## 2 Materials and methods

### 2.1 Materials

Middlebrook 7H11 agar, Middlebrook 7H9 broth, and OADC (oleic acid-albumin-dextrose-catalase) were purchased from Difco Laboratories (United States). Tween 80, lysozyme, and antibiotics (rifampicin, tetracycline, kanamycin) were procured from Sigma-Aldrich. RNeasy Mini Kit and UniPro GelEx/clean-up kit were obtained from Qiagen (Germany). Genomic DNA extraction kit, GoTaq green master mix, and nuclease-free water were from Promega. Oligonucleotides were synthesized by Sigma Chemical Co. (United States). SYBR green master mix and iScript™ cDNA synthesis kit were procured from Bio-Rad (Bio-Rad). Restriction enzymes, e.g., BamHI and HindIII were obtained from New England Biolabs. MEGAshortscript™ T7 transcription kit and Max™ Prehyb/hyb buffer solution were purchased from Invitrogen. Hybond-N+ nylon membrane was purchased from GE Healthcare. carbonyl cyanide *m*-chlorophenylhydrazone (CCCP) was purchased from Sigma Chemical Co. (United States). Plasmid pST-Ki was a generous gift from Dr. V. K. Nandicoori of the National Institute of Immunology, India. All other chemicals were of molecular biology grade.

### 2.2 Bacterial strain and growth conditions

The *M. tuberculosis* avirulent strain H37Ra was used as a model strain. The frozen glycerol stock was streaked on a Middlebrook 7H11 solid agar plate and stored at 37°C for 4–5 weeks. In a culture tube, one colony from the growing plate was inoculated with Middlebrook 7H9 broth (Difco Laboratories) supplemented with 10% OADC (oleic acid-albumin-dextrose-catalase; Difco Laboratories) and 0.05% (v/v) polysorbate 80 (Sigma-Aldrich). Subsequently, the cells were incubated at 37°C under shaking at 200 rpm. Cell growth while attaining OD_600_ 0.6–0.9, was regarded as the exponential growth phase of *M. tuberculosis*.

### 2.3 Antibiotic treatment

*M. tuberculosis* H37Ra cells were grown at 37°C in an orbital shaker incubator at 200 rpm until OD_600_ of the culture reached 0.9. The cells were then harvested at room temperature. After that, the cells were again suspended in 7H9 media and treated with rifampicin (2 μg/mL) for 30 min. Cells cultured under identical conditions without the addition of antibiotics were taken as control.

### 2.4 RNA isolation and RT-qPCR

Cell pellets with or without prior antibiotic treatment were re-suspended in 4 mL of PBS containing lysozyme (4 mg/mL). Lysozyme treatment was continued for 2 h at 37°C in a water bath with circulation. After that, cells were sonicated for 90 s (with alternate 10 s on or off pulse) using a Branson sonicator at 4°C. Total RNA was extracted using RNeasy Mini Kit (Qiagen). RNA concentration was measured using a JENWAY Genova Nanodrop spectrophotometer and RNA quality was assessed in a 1% agarose gel.

Total RNA (1 μg) from treated/untreated cells was used to synthesize cDNA using iScript^TM^ cDNA synthesis kit (Bio-Rad), along with random hexamer (Bio-Rad C1000, United States) as a primer. Complementary DNAs were diluted 100 times with nuclease-free water and 1 μL of diluted cDNA was employed in a quantitative real-time PCR reaction using SYBR green utilizing a CFX96 Touch™ Real-Time PCR detection system (Bio-Rad). A representative qPCR reaction of 20 μL contains 20 ng of cDNA, 1 μM of forward and reverse primers (P1 & P2 for MTS1338, P5 & P6 for 5S RNA, P7 & P8 for *cydC* mRNA in Supplementary Table S1), 10 μL of SYBR green master mix, and 8 μL of nuclease-free water mix. 5S RNA gene of *M. tuberculosis* was taken as an internal control. The 2^-ΔΔCt^ technique was used to calculate relative gene expression. Every experiment was carried out in compliance with the manufacturer’s guidelines.

### 2.5 Northern blot

Total RNA (15 μg), isolated from untreated or treated *M. tuberculosis* cells, were separated on 2% agarose-formaldehyde gel using 1X MOPS [3-(N-morpholino) propane sulfonic acid] buffer, which was then washed with deionized water. RNA was transferred from agarose gel to Hybond-N+ nylon membrane (GE Healthcare) by downward capillary transfer in 10X SSC buffer at room temperature overnight and UV crosslinked to the membrane by UV crosslinker (Herolab, Germany). A 5′-Cy5 labeled DNA probe (P11 for MTS1338 and P12 for 5S RNA in Supplementary Table S1) was used to detect MTS1338. Hybridization (Shake ‘n’ Stack™ Hybridization Chamber, Thermo Fisher Scientific, United States) of Cy5-labeled probe to crosslinked RNA on the membrane was promoted by incubating them in Northern Max™ Prehyb/hyb buffer solution (Invitrogen) overnight. The membrane was subsequently washed with pre-warmed SSC buffer three times and the bands were visualized by high-resolution gel imaging for the fluorescence system (G:Box Chemi-XX9, SYNGENE). Bands were quantitated by ImageJ.

### 2.6 Cloning and overexpression of MTS1338

The genomic DNA of *M. tuberculosis* H37Ra cells was extracted using the Genomic DNA Extraction Kit (Promega Co., United States). The gene encoding MTS1338 was amplified from genomic DNA in a PCR reaction using KOD hot start polymerase (Invitrogen) with primers P3 and P4 listed in Supplementary Table S1. The amplicon was purified by gel extraction (Qiagen). Both the PCR products and the shuttle vector pST-Ki were digested with BamHI and HindIII for 2 h at 37°C. Digested products were purified through phenol: chloroform: isoamyl alcohol (25, 24:1) extraction and were ligated by T4 DNA ligase (New Eng Biolab). The resulting recombinant plasmids (pMTS1338) were transformed into *Escherichia coli* DH5α cells, which were grown on an LB (Kan+) plate overnight at 37°C. Cloning of gene encoding MTS1338 under the control of tetracycline promoter was confirmed by DNA sequencing. Recombinant pMTS1338 from *E. coli* cells was electroporated into *M. tuberculosis* H37Ra cells and subsequent MTS1338 overexpression was monitored upon the addition of tetracycline (1 μg/mL) for 72 h.

### 2.7 Growth analysis

The 7H9 medium supplemented with 10% OADC and 0.05% Tween 80 was used to grow the mycobacterial bacilli. Carbonyl cyanide 3-chlorophenylhydrazone (4 μg/mL) was added to the cells 5 days after the cells (OD_600_-0.9) were treated with rifampicin (2 μg/mL). The cells were continued to grow for 16 days under shaking (200 rpm) at 37°C. Growth was monitored by measuring the optical density of the culture at 600 nm every 24 h with a UV spectrophotometer (JASCO).

### 2.8 RNA decay

Overnight cultures were diluted 1:100 into fresh 7H9 medium supplemented with 10% OADC and 0.05% Tween 80 and grown to exponential phase (OD_600_-0.9) followed by treatment with 500 μg/mL rifampicin (Chen et al., 2019). Equal volumes of cells were removed for the zero-time sample after 1 min of rifampicin addition to allow complete inhibition of new transcription and immediately mixed with ice-cold stop solution (95% ethanol, 5% phenol). Additional samples were removed at indicated time points in the figure legends. Changes in *cydC* mRNA accumulation were determined by RT-qPCR analysis.

### 2.9 *In vitro* transcription

Genomic DNA of *M. tuberculosis* H37Ra cells was used as a template for the synthesis of genes of CydC and MTS1338 by PCR amplification using primers (P8 & P14 for *cydC*, P2 & P13 for MTS1338 and P10 & P11 for *katG* mRNA) listed in Supplementary Table S1. The forward primer was fused with a promoter sequence of T7 RNA polymerase. Templates were gel extracted using a QIAquick® gel extraction kit. Each template of 100 nM was added in an *in vitro* transcription reaction using a MEGAshortscript™ T7 transcription kit. Transcripts were extracted with phenol: chloroform: isoamyl alcohol (25: 24: 1) and precipitation overnight with ethanol at-20°C. The concentration of RNA was estimated spectrophotometrically by NanoDrop® spectrophotometer (Maestrogen, Taiwan) and RNA quality was checked on 6% polyacrylamide, 7.5 M urea gel.

### 2.10 Electrophoretic mobility shift assay

Transcripts of MTS1338 and *cydC* mRNA fragment (916–1,030 residues) were denatured at 80°C for 10 min and then cooled on ice. Binding reactions (10 μL) were conducted by incubating cydC mRNA (10 nM) with increasing amounts of MTS1338 (0–8 μM) at 37°C for 30 min in 1X binding buffer [100 mM Tris acetate pH 7.6, 500 mM NaOAc, and 25 mM Mg (OAc)_2_]. Aliquots of each binding reaction were separated in an agarose gel (2%) using 1X MOPS buffer at 4°C. Gel was stained with 1X SYBR™ Gold Nucleic Acid Gel Stain (Invitrogen) and bands were visualized by high-resolution gel imaging for the fluorescence system (G: Box Chemi-XX9, SYNGENE).

### 2.11 EtBr accumulation assay

H37Ra cells harboring empty pST-Ki or recombinant pMTS1338 plasmid were grown in 7H9 medium supplemented with 10% OADC until the mid-exponential phase (OD_600_-0.9). Cells were then treated with tetracycline (1 μg/mL) for 72 h for the extrachromosomal induction of MTS1338 followed by the addition of EtBr (1 μg/mL). After 10 min of EtBr exposure at 37°C, cells were treated with an efflux pump inhibitor CCCP with a final concentration of 6.25 μM. Cells were then pelleted down by centrifugation at 3500 g for 5 min and then resuspended in 1 mL of Phosphate buffer saline with/without CCCP. Intracellular accumulation of EtBr was measured *in situ* by fluorimetry with excitation at 520 nm and emission at 590 ± 20 nm. Fluorescence was monitored every 20 min, and the measurements were continued for 60 min.

### 2.12 Statistical analysis

Data obtained from experimental studies were presented as mean ± standard deviation. One-way analysis of variance was conducted, and a difference of *p* < 0.05 was considered statistically significant.

## 3 Results

### 3.1 MTS1338 is upregulated following rifampicin treatment

**Figure 1:**
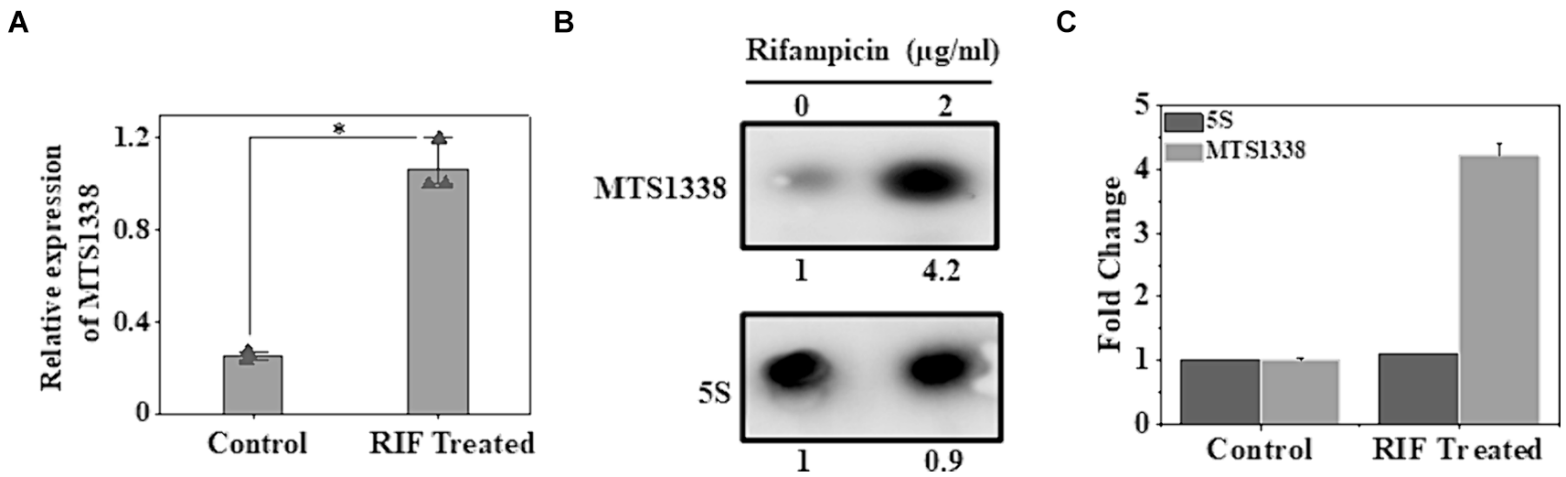
Effect of rifampicin **(A)** treatment on the intracellular abundance of MTS1338 in *Mycobacterium tuberculosis* cells. Mid-exponentially grown cells (OD_600_–0.9) were exposed to rifampicin (2 μg/mL) and then were harvested. Total RNA was isolated and MTS1338 abundance was estimated by RT-qPCR analysis. 5S gene was taken as an internal control. The relative amounts of the transcripts are presented as the average ± standard deviation (SD) from three separate experiments and normalized to the level of the untreated sample. One-way ANOVA was conducted and a difference of *p < 0.05 was considered statistically significant. **(B)** Total RNA (15 μg) isolated from the cells treated with/without rifampicin (2 μg/mL) were separated in a 2% agarose formaldehyde gel and analyzed by northern blot. The number under each lane represents the relative amount of MTS1338 when compared to untreated sample, which was set as 1. 5S rRNA gene was taken as a loading control. Northern blots were carried out three times and one representative blot is presented. **(C)** Northern blot experiments were carried out three times with 5′-Cy5 labeled DNA oligonucleotide probes for RNAs. Bands were quantitated by ImageJ. The amount of MTS1338 and 5S RNA in the untreated cell was set at 1.

H37Ra cells were used as a model strain for our experiments as both strains of *M. tuberculosis*, H37Ra, and H37Rv exhibit equivalent minimum inhibitory concentrations to most antituberculosis drugs (Heinrichs et al., 2018). Given the induction of MTS1338 in response to multiple infection-associated stresses and its regulation to remodel the global gene expression of *M. tuberculosis*, it insinuated that MTS1338 might play some role in mycobacterial adaptation to antibiotic treatment. To address that hypothesis *M. tuberculosis* H37Ra cells were grown in Middlebrook 7H9 broth supplemented with 10% OADC and 0.05% (v/v) polysorbate 80, until OD_600_ reached 0.9 and then treated with rifampicin (2 mg/mL), a frontline antimycobacterial drug, for 30 min at 37°C under shaking condition. The concentration of rifampicin was used in a similar experiment as described (Lake et al., 2023). Another *M. tuberculosis* culture identically treated except for rifampicin was considered as a control. MTS1338 abundance in the total RNA isolated from rifampicin-treated and untreated cells was measured by RT-qPCR analysis. Figure 1A clearly shows that cells accumulate as much as ~5-fold more MTS1338 in 30 min following rifampicin treatment. In addition, northern blotting for MTS1338 in the total RNA isolated from rifampicin-treated or control cells revealed a 4-fold more accumulation of MTS1338 in rifampicin-treated cells (Figures 1B,C) than in the control untreated cells. These observations lead to the conclusion that MTS1338 is a rifampicin-induced sRNA that is potentially involved in adaptation to rifampicin-treated conditions.

### 3.2 Enhanced intracellular MTS1338 abundance increases the growth rate in the presence of rifampicin

**Figure 2:**
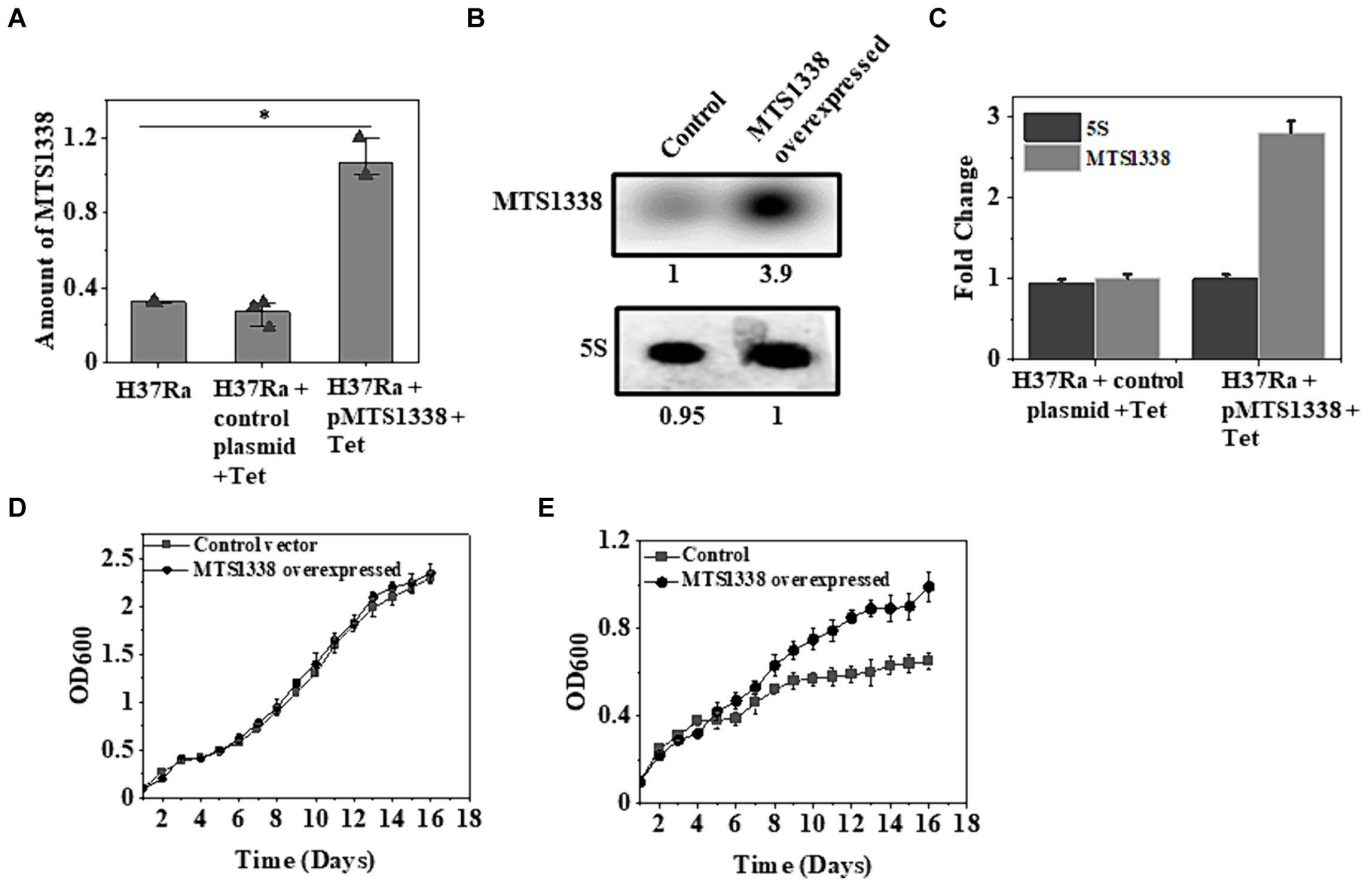
A higher MTS1338 abundance increases the growth rate in the presence of rifampicin. **(A)** Tetracycline (1 μg/mL) was added to the exponentially grown *M. tuberculosis* cells (OD_600_ = 0.9) harboring empty or recombinant pMTS1338 plasmids. Cells continued to grow for 72 h and the total RNA was extracted from each sample. MTS1338 abundance was estimated by RT-qPCR analysis. 5S gene was taken as an internal control. The relative amounts of the transcripts are presented as the average ± SD from three separate experiments and normalized to the level of the untreated sample. One-way ANOVA was conducted and a difference of *p < 0.05 was considered statistically significant. **(B)** Northern blot was carried out using 15 μg of total RNA isolated from the cells with/without overexpressing MTS1338. 5S RNA gene was taken as loading control. Northern blot experiments were carried out three times and identical results were obtained. A representative of Northern blots was presented. The number under overexpressed lane represents the relative amount of RNA when compared with control lane, which was set 1.0. **(C)** Bands of three independent northern blots were quantitated by ImageJ. Data were presented as the average ± SD. The amount of MTS1338 and 5S RNA in the untreated cell was set at 1. **(D)** H37Ra cells harboring recombinant pMTS1338 plasmid or empty vector were grown at 37°C. OD_600_ was monitored in regular interval and was plotted against time. The data presented was the average ± S.D. of three independent experiments. Growth experiments were also carried out in presence of rifampicin (5 μg/mL) added at the day 1 **(E)**.

As MTS1338 accumulates to a higher degree following antibiotic treatment on *M. tuberculosis* cells, it was of immense interest to investigate whether overexpression of MTS1338 ameliorates the growth rate of the cells. To address that question, the gene encoding MTS1338 was cloned in the pST-Ki shuttle vector under the control of *P_tet_* promoter, and the recombinant plasmid thus obtained (pMTS1338) was electroporated in *M. tuberculosis* H37Ra cells. Overexpression of MTS1338 was monitored by adding tetracycline (1 μg/mL) to the culture media for 72 h. Estimation of intracellular MTS1338 abundance upon addition of tetracycline resulted in ~3.5-fold more accumulation of MTS1338 when compared to the control cells analyzed by RT-qPCR (Figure 2A). Northern blot analysis (Figure 2B) of MTS1338 in the total RNA isolated from the cells with/without MTS1338 overexpression also substantiated ~4-fold more accumulation of MTS1338 in the cells harboring pMTS1338 plasmid (Figure 2C).

To examine whether higher MTS1338 abundance affects cell growth or not, we monitored the growth of cells with/without extrachromosomal MTS1338 overexpression and found that overexpression of MTS1338 in H37Ra cells under normal conditions did not show any alteration of growth rate (Figure 2D). This fact indicated that a higher accumulation of MTS1338 is not detrimental to cell viability. However, growth analysis in the presence of rifampicin revealed a significant difference in the growth rate of the cells with or without MTS1338 overexpression (Figure 2E). H37Ra cells overexpressing MTS1338 appeared to grow at a higher rate with a generation time of 6 days than the cells containing empty vector (generation time 9 days). This firmly established that MTS1338 promotes cell growth in the presence of first-line antibiotic rifampicin.

### 3.3 Elevated MTS1338 induces the gene encoding efflux protein CydC

**Figure 3:**
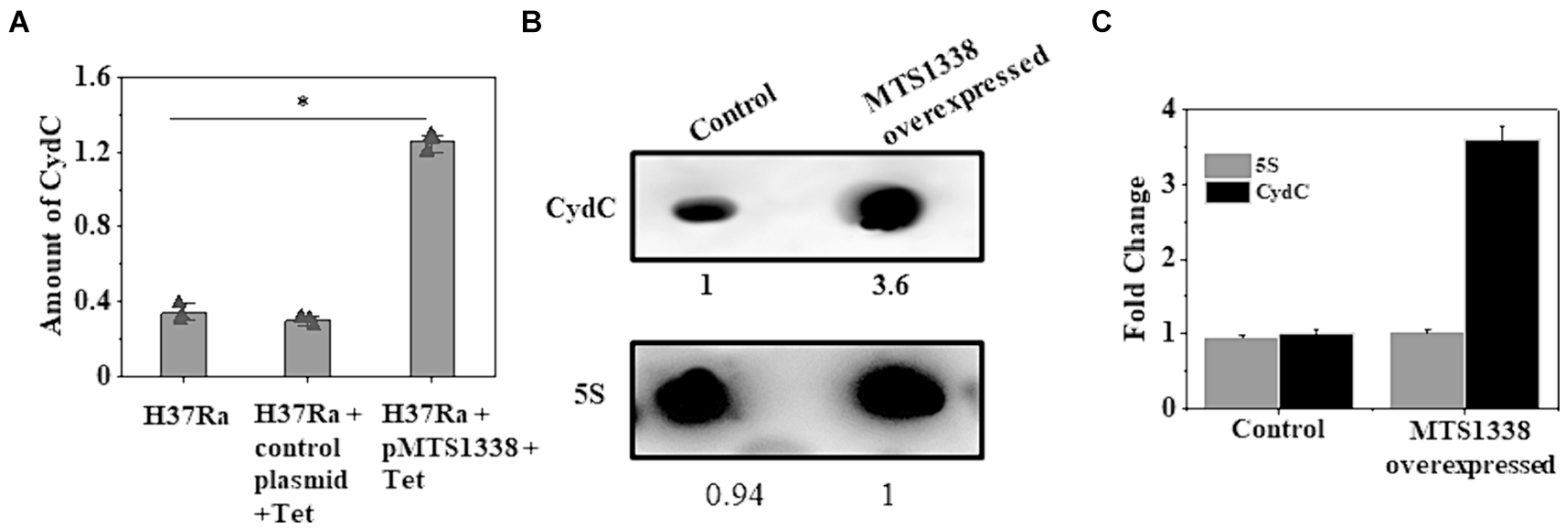
**(A)** Relative expression of *cydC* mRNA was estimated by RT-qPCR analysis in the exponentially grown *M. tuberculosis* cells (OD_600_ = 0.9) harboring control or recombinant pMTS1338 vectors. 5S gene was taken as an internal control. The relative amounts of *cydC* mRNA are presented as the average ± standard deviation from three separate experiments and normalized to the level of the untreated sample. One-way ANOVA was conducted and a difference of *p < 0.05 was considered statistically significant. **(B)** Northern blot was carried out using 5’-Cy5-labeled DNA probe to detect *cydC* mRNA present in the total RNA isolated from the cells with/without overexpressing MTS1338. 5S was taken as a loading control. A representative of three independent northern blots with identical results was presented. The number under each lane represents the relative amount of *cydC* when compared with control. **(C)** Bands of three independent northern blots were quantitated by ImageJ. Data were presented as the average ± SD. The amount of *cydC* and 5S RNA in the control cell was set at 1.

MTS1338-driven increased cellular robustness in opposition to rifampicin apprised us to investigate how MTS1338 acts to relieve the antagonistic effect created by rifampicin. To address that question, we aimed to examine the expression of genes encoding ABC transporters since it is one of the most predominant intrinsic mechanisms by which *M. tuberculosis* can ride out antibiotic treatment is drug efflux (Remm et al., 2022). The mycobacterial *cydC* gene, transcribed from the *cydABDC* gene cluster, encodes an efflux protein. The absence of CydC in *M. tuberculosis* alleviates its ability to survive the transition from acute to chronic infection. Expression of *cydABDC* has also been shown to be induced by hypoxia and NO stress (Shi et al., 2005). We selected *cydC* gene for our investigation of whether MTS1338 exerts any effect on *cydDC* expression as in many instances *cydDC* transporters are recognized to help persist *M. tuberculosis* against drugs like isoniazid (8). To settle that hypothesis, we compared the expression of *cydC* gene in H37Ra cells harboring either pMTS1338 or empty pST-Ki vectors and found >3-fold induction of *cydC* in MTS1338 overexpressing cells (Figure 3A). Northern blot analysis (Figure 3B) for the determination of *cydC* accumulation revealed that the cells with higher MTS1338 abundance upregulated *cydC* transcripts by >3-fold (Figure 3C), which reinforced RT-qPCR data. These experiments pointed to the involvement of MTS1338 in inducing the *cydC* gene.

**Figure 4:**
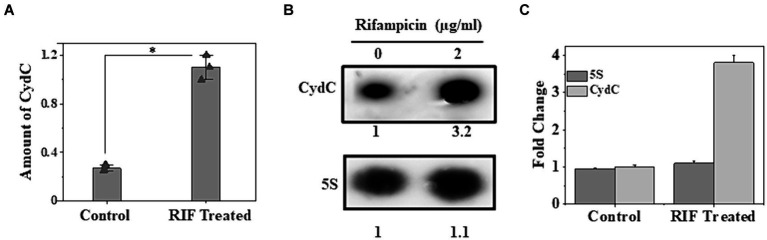
Accumulation of *cydC* in *M. tuberculosis* while treated with rifampicin (2 μg/mL). **(A)** Mid-log phase H37Ra cells (OD_600_ = 0.9) were exposed to antibiotics for 30 min at 37°C. Relative amount of *cydC* in the total RNA was measured by RT-qPCR analysis. 5S gene was taken as an internal control. The relative amounts of *cydC* mRNA are presented as the average ± standard deviation from three separate experiments and normalized to the level of the untreated sample. One-way ANOVA was conducted and a difference of *p < 0.05 was considered statistically significant. **(B)** Total RNA (15 μg) from rifampicin treated and untreated samples were separated in in a 2% agarose formaldehyde gel and analyzed by northern blot. The number under each lane represents the relative amount of *cydC* when compared to untreated sample, which was set as 1. 5S RNA gene was taken as a loading control. Northern blots were carried out three times and one representative blot is presented. **(C)** Northern blot experiments were carried out three times with 5′-Cy5 labeled DNA oligonucleotide probes for RNAs. Bands were quantitated by ImageJ. The amount of MTS1338 and 5S RNA in the untreated cell was set at 1.

Upregulation of *cydC* in response to higher intracellular MTS1338 abundance and enhanced accumulation of MTS1338 following antibiotic treatment instigated us to investigate whether *cydC* is also upregulated in response to antibiotic treatment. To examine that objective, we compared the intracellular *cydC* levels in the cells treated with/without rifampicin by RT-qPCR and northern blot analysis. It is conspicuous from Figure 4A that *cydC* accumulation analyzed through RT-qPCR was induced by ~4-fold following rifampicin treatment. An analogous result of rifampicin-driven *cydC* induction was obtained through northern blot analysis (Figures 4B,C). Investigation of the expression of *cydD*, which encodes a different efflux protein, revealed that rifampicin treatment did not show any significant effect on its intracellular abundance in *M. tuberculosis* (Singh and Dutta, unpublished observation). These experiments firmly established that MTS1338 has a profound effect in maintaining intracellular *cydC* levels against antibiotic treatment.

### 3.4 MTS1338 directly interacts with the coding region of *cydC* mRNA

**Figure 5:**
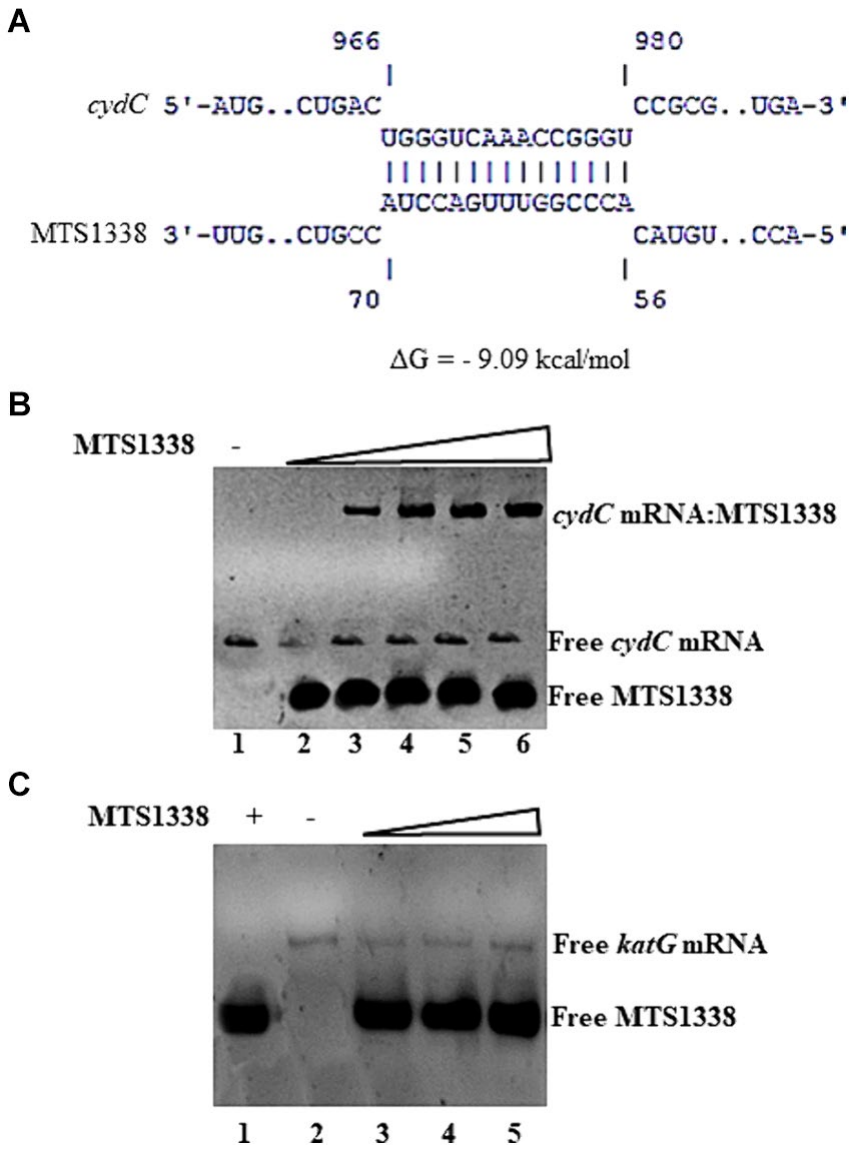
**(A)** Base pairing region between MTS1338 and *cydC* mRNA fragment as predicted by IntaRNA program. The numbering of nucleotides on MTS1338 and *cydC* mRNA is relative to the transcription start site of each RNA. **(B,C)** Interaction between MTS1338 and *cydC* mRNA **(B)** and *katG* **(C)**. Fragment of *cydC* and *katG* mRNA (10 pmol) was incubated with 10, 20 and 30 pmol of MTS1338 (lanes 4, 5, and 6 respectively) in a 20 μL reaction. Numbers at the bottom of the gels represent the lanes **(C)**.

MTS1338-mediated upregulation of *cydC* mRNA brought up the question of whether MTS1338 regulates *cydC* abundance by directly interacting with it. To address that question, both *cydC* mRNA and MTS1338 sequences were used in the IntaRNA (Mann et al., 2017) to identify if any complementary region(s) exists between them. A region of 15-nt on *cydC* mRNA (966–980 residues) was found to be completely complementary to an intermediate portion (56–70 residues) of MTS1338 (Figure 5A). To confirm that hypothesis, both MTS1338 RNA and *cydC* mRNA fragments surrounding residues 916 to 1,030 that possesses region complementary to MTS1338 were transcribed and purified *in vitro*. Incubation of MTS1338 with *cydC* fragment resulted in the formation of *cydC*-MTS1338 binary complex, which was observed as a high molecular weight band generated in the gel-shift assay (Figure 5B). Addition of increasing amounts of MTS1338 to a constant amount of *cydC* mRNA gave rise to a concomitant increase in the population of *cydC*-MTS1338 binary complex. This experiment confirmed that MTS1338 directly interacts with the coding region of *cydC* mRNA potentially stabilizing it at the posttranscriptional level. A control experiment with *katG* mRNA did not show any interaction with MTS1338 (Figure 5C).

### 3.5 MTS1338 overabundance increases the stability of *cydC* mRNA

**Figure 6:**
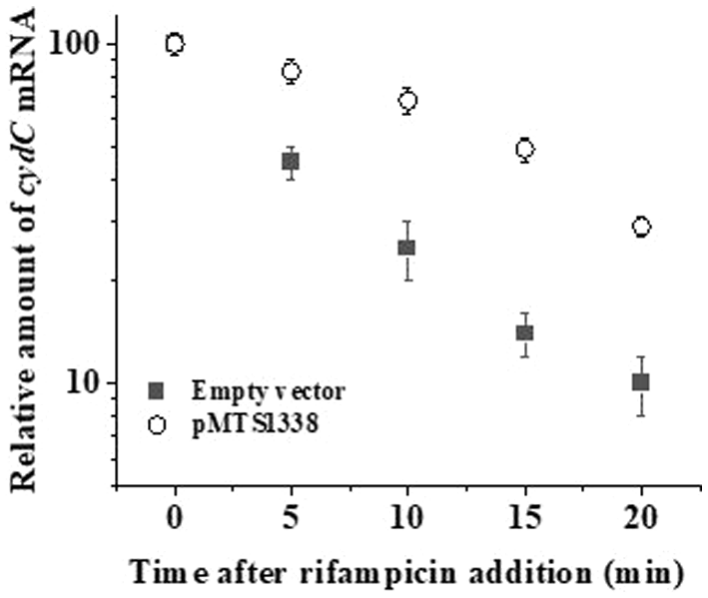
Stability of *cydC* mRNA with/without MTS1338 overexpression. Cells harboring either empty or pMTS1338 plasmids were grown to exponential phase (OD_600_–0.9) and then tetracycline (1 μg/mL) was added to cells. Cells were further grown for 72 h and were treated with 500 mg/liter rifampicin. Samples were withdrawn at the indicated time points and the amount of cydC mRNA was determined by RT-qPCR analysis.

Direct interaction of MTS1338 to the coding region of *cydC* mRNA raised the question of whether MTS1338 binding to *cydC* mRNA increased its stability *in vivo*. To help answer this question, we first overexpressed MTS1338 in exponentially growing cells (OD_600_–0.9) and subsequently rifampicin (500 μg/mL) was added to it. The use of this high concentration of rifampicin completely inhibits new transcription. The amount of *cydC* mRNA in the cells with/without overexpressing MTS1338 was measured at different time points after rifampicin addition by RT-qPCR analysis. As can be seen in Figure 6, overexpression of MTS1338 led to dramatic stabilization of the *cydC* message, increasing its half-life from ~4 min to ~15 min. This firmly indicated that the binding of MTS1338 to *cydC* mRNA protected from intracellular degradation possibly by ribonucleases.

### 3.6 A higher accumulation of MTS1338 increases rifampicin efflux from axenically grown *Mycobacterium tuberculosis*

**Figure 7:**
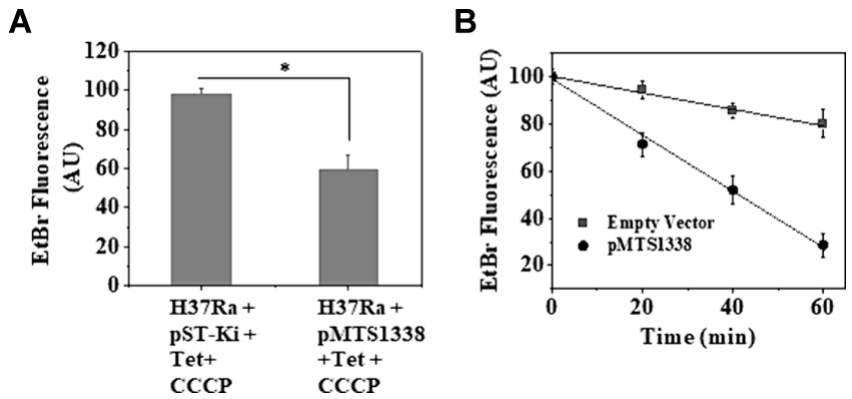
**(A)** Accumulation of EtBr in *M. tuberculosis* cells with/without MTS1338 overexpression. Cells (OD_600_ = 0.9) containing empty or recombinant pMTS1338 plasmids were allowed to grow in presence of tetracycline (1 μg/mL) for 72 h followed by the addition of EtBr (1 μg/mL). After 10 min CCCP (6.25 μM) was added to the culture. Cells were then pelleted down and resuspended in PBS containing CCCP. Intracellular EtBr fluorescence was measured by fluorimetry. Data was presented as the average ± SD from three separate experiments. One-way ANOVA was conducted and a difference of *p < 0.05 was considered statistically significant. **(B)** After the treatment with EtBr and CCCP, cells were resuspended in PBS and intracellular accumulation of EtBr was measured by fluorimetry at indicated time points. Data was presented as the average ± SD from three separate experiments.

We measured intrabacterial ethidium bromide (EtBr) accumulation as a substitute for rifampicin efflux as EtBr has been considered as an artificial efflux pump substrate (Lake et al., 2023). The fluorescence of EtBr increases by ~20-fold when intercalated with DNA (Jernaes and Steen, 1994). Thus, occluding EtBr efflux from *M. tuberculosis* cells by efflux pump inhibitor renders it more fluorescent, which can be measured by spectrofluorimetry. To examine the effect of a higher accumulation of MTS1338 on rifampicin efflux, *M. tuberculosis* cells with/without overexpressing MTS1338 were exposed to EtBr for 10 min at 37°C and the efflux pump inhibitor CCCP was subsequently added to the cells. Cells were then centrifuged and resuspended in PBS and CCCP was added to the resuspended cells. Figure 7A shows the accumulation of EtBr in *M. tuberculosis* with/without overexpressing MTS1338 treated with CCCP. It is evident in Figure 7A that cells with a higher intracellular MTS1338 abundance accumulated as much as 50% less EtBr than what was obtained in the cells with a low abundance of MTS1338. This experiment firmly suggested that a higher abundance of MTS1338 promoted a lower accumulation of EtBr potentially deporting it from the cells.

In a similar line, to investigate the rate of EtBr efflux in cells with empty/recombinant pMTS1338 plasmids, EtBr-containing cells were treated with CCCP and then resuspended in PBS to substantially diminish the inhibitory effect of CCCP on efflux proteins. After that intracellular EtBr accumulation was measured by spectrofluorimetry every 20 min up to 60 min. Figure 7B shows that the decrease in the inhibitory effect of CCCP resulted in the outflow of EtBr from the cells, which was reflected by the concomitant decrease in EtBr fluorescence with increasing time. Interestingly, a nearly 75% decrease in EtBr fluorescence was observed for the cells with higher MTS1338 abundance, whereas for the cells with low intracellular MTS1338 population, only a ~ 20% decrease in EtBr fluorescence was measured after 60 min. This experiment established that MTS1338 facilitates the deportation of EtBr, a surrogate of rifampicin, from the cells.

## 4 Discussion

Although *M. tuberculosis* possesses an array of eccentric mechanisms to withstand antibiotic action, sRNA-mediated drug tolerance of mycobacteria has not been reported earlier. In the current study, we systematically analyzed the remodeling of an intracellular regulatory circuit of *M. tuberculosis* in response to antibiotic treatment. MTS1338, a distinctive sRNA exists only in pathogenic mycobacterial strains (Arnvig et al., 2011) and can confer pathogenic properties to nonpathogenic mycobacteria while expressed in a heterologous manner (Bychenko et al., 2021). Treatment of first-line antitubercular drug rifampicin on *M. tuberculosis* upregulated MTS1338, which in turn was found to ameliorate its survival in the presence of the drug. The treatment of a second-line drug kanamycin on *M. tuberculosis* also showed an identical effect on MTS1338. Examination of how MTS1338 promotes *M. tuberculosis* survival under rifampicin-exposed conditions revealed that a higher MTS1338 abundance induced an efflux protein CydC that minimizes drug concentration in the cell.

The global emergence of pathogens resistant to antimicrobial agents accounts for the increasing health crisis of people. Multifaceted approaches for the mitigation of disease prevalence by promoting antibiotic stewardship are required to control antimicrobial resistance and tolerance. Understanding how pathogens remodel their gene regulatory pathways against the existing antibiotic arsenal is a critical step for the development of new antimicrobial drugs or improving the self-life of current antibiotics (Mediati et al., 2021). Although multiple mechanisms have been elucidated for mycobacterial resistance against antibiotics, a small RNA regulator contributing to antimycobacterial resistance has never been reported before this study. However, the instances of sRNAs exist in pathogens other than mycobacteria acting as regulatory hubs for antibacterial resistance. A highly conserved sRNA SprX in *Staphylococcus aureus* causing bacteraemia, infective endocarditis, and osteomyelitis in humans alters antibiotic sensitivity by negatively regulating transcription factor SpoVG (Mediati et al., 2021; Eyraud et al., 2014; Liu et al., 2016). Two sRNAs, Sr0161 and ErsA, in multidrug-resistant *Pseudomonas aeruginosa* negatively regulate a major outer-membrane porin OprD, which is responsible for the uptake of carbapenem antibiotic. Thus, sRNA-mediated downregulation of OprD leads to bacterial resistance against carbapenem (Ochs et al., 1999; Zhang et al., 2017). Numerous sRNA in *E. coli*, e.g., MicF, GcvB, RyhB, etc. have been shown to modulate antibiotic sensitivity leading to resistance (Dersch et al., 2017). Many other pathogens like *Salmonella* sp. (Acuña et al., 2016), *Vibrio* sp. (Peschek et al., 2020), etc. exploit sRNAs to develop resistance against antibiotics. MTS1338-mediated growth advantage of *M. tuberculosis* under antibiotic-treated conditions confirms the sRNA orchestration of *cydC* upregulation for mycobacterial adaptation to antibiotic treatment.

Efflux of drugs through membrane transporters or efflux pump proteins is one of the paramount strategies of *M. tuberculosis* for acquiring antibiotic resistance (10). Efflux pumps are membrane-spanning proteins primarily controlled by regulatory systems that have evolved to counter antibiotics (Smith et al., 2013). In the current context, MTS1338 after being induced by rifampicin acts as a pivot that upregulates a higher intracellular abundance of efflux proteins CydC thereby promoting the deportation of drugs leading to the improvement of cellular fitness (Andersson and Hughes, 2010). Thus, CydC activity in *M. tuberculosis* is coupled to MTS1338 regulation and is conceivable to be involved during mycobacterial infection owing to MTS1338’s role under that condition. *M. tuberculosis* possesses at least 18 different transporters related to antibiotic susceptibility (Viveiros et al., 2012). Thus, it would not be surprising if MTS1338 controls multiple transporters in response to antibiotic treatment. An example lies in *M. tuberculosis*, as a transcription regulator Lsr2 in this bacterium being upregulated by isoniazid and ethambutol negatively controlled the expression of two transporters IniBAC and EpfA, which led to obtaining resistance against antibiotics (Colangeli et al., 2007). CydC also can be controlled by multiple regulators as it happens in *E. coli* where three regulatory systems for antibiotics controlled a single transporter protein AcrB, a major determinant for multidrug resistance (Alekshun and Levy, 1997). Additional studies are required to unfold the MTS1338-driven regulatory processes how MTS1338 facilitates the outflow of antibiotics through CydC.

Given the multidimensional role of MTS1338, it would not be surprising if MTS1338 is accountable for turning on other pathways leading to antimycobacterial resistance. As MTS1338 is induced in response to both front-line and second-line drugs, it is conceivable that MTS1338 might promote antibacterial resistance against a broad range of antibiotics. Several sigma factors and WhiB protein transcription factors have been identified to be responsible for antibiotic susceptibility (Miotto et al., 2022). The key among the WhiB proteins that promote antibacterial drug resistance is WhiB7 (Burian et al., 2013; Reeves et al. 2013; Pisu et al., 2017). WhiB7 is upregulated by several 100-folds upon exposure to front-line drugs like rifampicin and is barely controlled by MTS1338 as increased intracellular abundance does not significantly alter the intracellular accumulation of *whiB7* transcript (Singh and Dutta, unpublished observation). Likewise, SigE in *M. tuberculosis* was reported to be one of the leading among 13 Sigma factors gaining resistance against antibiotics (Pisu et al., 2017). *M. tuberculosis* also exploits many other transcription factors like GntR, XRE, etc. to obtain resistance (Miotto et al., 2022). Additional work is required to establish whether MTS1338 is involved in all the abovementioned mechanisms.

The current study depicted a novel antimycobacterial resistance mechanism regulated by a distinctive sRNA MTS1338 in *M. tuberculosis*. This adds substantially to our knowledge of how *M. tuberculosis* triggers a regulatory cascade for acquiring resistance against antimycobacterial drugs controlled by a sRNA, which was never unveiled before.

## Data Availability

The original contributions presented in the study are included in the article/Supplementary material, further inquiries can be directed to the corresponding author.
