# Supplementary material for: A virulence-associated small RNA MTS1338 activates an ABC transporter CydC for rifampicin efflux in Mycobacterium tuberculosis
Source: Front Microbiol. 2024 Sep 19;15:1469280. doi: 10.3389/fmicb.2024.1469280 (PMC11446857; doi:10.3389/fmicb.2024.1469280)
Supplement: Supplementary file 1 [file Table_1.DOCX]

**A virulence-associated small RNA MTS1338 activates an ABC transporter CydC for rifampicin efflux in *Mycobacterium tuberculosis***

**Saumya Singh^1^ and Tanmay Dutta^1^***

^1^RNA Biology Laboratory, Department of Chemistry, Indian Institute of Technology Delhi, Hauz Khas, New Delhi 110016.

**Table S1**: Oligonucleotide and probe use in this study.

| Oligos | Sequence (5′ to 3′) | Used for |
| --- | --- | --- |
| MTS1338 Forward (P1) | GGAAACCCGGTGATCTGCCC | RT-qPCR |
| MTS1338 Reverse (P2) | GATCTGCCCGAAGGC GGTAG | RT-qPCR |
| MTS1338 Forward  (P3) | AAA GGATCC GGAAACCCGGTGATCTGCCC | Cloning |
| MTS1338 Reverse  (P4) | AAA TTCGAA GATCTGCCCGAAGGCGGTAG | Cloning |
| MTB 5S Forward (P5) | TTACGGCGGCCACAGC | RT-qPCR |
| MTB 5S Forward (P6) | CTAGGACACACCGCCG | RT-qPCR |
| *cydC* Forward (P7) | CGACCGAGTGCTGTGAGTC | RT-qPCR |
| *cydC* Reverse (P8) | GCCAGTCGCTCGCAGTAG | RT-qPCR |
| *katG* Forward (P9) | TAATACGACTCACTATAGGGAGAGTCATCTACTGGGGTCTATG | EMSA |
| *katG* Reverse (P10) | GTACCTTCAGATTGAGCCGGTTGGG | EMSA |
| MTS1338 (P11) | Cy5-AACAGGATGAGGACTAGCCCGAAGGCGGAT | Northern |
| MTB 5S (P12) | Cy5-GC CCA CCT TTT CAT CCT GTG GCG GCTTGT | Northern |
| MTS1338 Forward (P13) | TAATACGACTCACTATAGAAAAAACCGGGGAAACCCGGTGA | EMSA |
| *cydC* Forward (P14) | TAATACGACTCACTATAGGGAGAGATGCGCTCGACGCCGCCGCACGAACG | EMSA |
